# Expanding the molecular and phenotypic spectrum of truncating *MT-ATP6* mutations

**DOI:** 10.1212/NXG.0000000000000381

**Published:** 2020-01-07

**Authors:** Enrico Bugiardini, Emanuela Bottani, Silvia Marchet, Olivia V. Poole, Cristiane Beninca, Alejandro Horga, Cathy Woodward, Amanda Lam, Iain Hargreaves, Annapurna Chalasani, Alessandra Valerio, Eleonora Lamantea, Kerrie Venner, Janice L. Holton, Massimo Zeviani, Henry Houlden, Rosaline Quinlivan, Costanza Lamperti, Michael G. Hanna, Robert D.S. Pitceathly

**Affiliations:** Department of Neuromuscular Diseases (E. Bugiardini, O.V.P, A.H., H.H., R.Q., M.G.H., R.D.S.P.), UCL Queen Square Institute of Neurology and The National Hospital for Neurology and Neurosurgery, London, United Kingdom; Mitochondrial Medicine Group (E. Bottani, C.B., M.Z.), Medical Research Council Mitochondrial Biology Unit, Cambridge, United Kingdom; Department of Molecular and Translational Medicine (E. Bottani, A.V.), University of Brescia; Medical Genetics and Neurogenetics Unit (S.M., E.L., C.L.), Fondazione IRCCS Istituto Neurologico, “C. Besta,” Milan, Italy; Neurogenetics Unit (C.W.), and Neurometabolic Unit (A.L., I.H., A.C.), The National Hospital for Neurology and Neurosurgery; Division of Neuropathology (K.V., J.L.H.), UCL Queen Square Institute of Neurology; and Dubowitz Neuromuscular Centre (R.Q.), Great Ormond Street Hospital, London, United Kingdom.

## Abstract

**Objective:**

To describe the clinical and functional consequences of 1 novel and 1 previously reported truncating *MT-ATP6* mutation.

**Methods:**

Three unrelated probands with mitochondrial encephalomyopathy harboring truncating *MT-ATP6* mutations are reported. Transmitochondrial cybrid cell studies were used to confirm pathogenicity of 1 novel variant, and the effects of all 3 mutations on *ATPase 6* and complex V structure and function were investigated.

**Results:**

Patient 1 presented with adult-onset cerebellar ataxia, chronic kidney disease, and diabetes, whereas patient 2 had myoclonic epilepsy and cerebellar ataxia; both harbored the novel m.8782G>A; p.(Gly86*) mutation. Patient 3 exhibited cognitive decline, with posterior white matter abnormalities on brain MRI, and severely impaired renal function requiring transplantation. The m.8618dup; p.(Thr33Hisfs*32) mutation, previously associated with neurogenic muscle weakness, ataxia, and retinitis pigmentosa, was identified. All 3 probands demonstrated a broad range of heteroplasmy across different tissue types. Blue-native gel electrophoresis of cultured fibroblasts and skeletal muscle tissue confirmed multiple bands, suggestive of impaired complex V assembly. Microscale oxygraphy showed reduced basal respiration and adenosine triphosphate synthesis, while reactive oxygen species generation was increased. Transmitochondrial cybrid cell lines studies confirmed the deleterious effects of the novel m.8782 G>A; p.(Gly86*) mutation.

**Conclusions:**

We expand the clinical and molecular spectrum of *MT-ATP6*-related mitochondrial disorders to include leukodystrophy, renal disease, and myoclonic epilepsy with cerebellar ataxia. Truncating *MT-ATP6* mutations may exhibit highly variable mutant levels across different tissue types, an important consideration during genetic counseling.

Mitochondrial disorders are genetic diseases caused by mutations in mitochondrial DNA (mtDNA)-encoded or nuclear-encoded genes; the protein products of which are essential for adenosine triphosphate (ATP) synthesis by oxidative phosphorylation (OXPHOS). ATP is generated from adenosine diphosphate and inorganic phosphate by mitochondrial ATP synthase (OXPHOS complex V), which harnesses the proton electrochemical gradient generated across the inner mitochondrial membrane by the sequential transfer of electrons across the mitochondrial electron transport chain enzymes (complexes I–IV).^1^ ATP synthase comprises 16 subunits, 14 nuclear-encoded and 2 mtDNA-encoded (*MT-ATP6/8*). Numerous pathogenic mutations in *MT-ATP6/8* are reported. The most common of these is the pathogenic m.8993T>G/C mutation in *MT-ATP6*, encoding the *ATP6* subunit of mitochondrial ATP synthase, which is proven to both disrupt assembly of complex V and reduce catalytic activity of the enzyme.^2^ Classic mitochondrial phenotypes described with *MT-ATP6* mutations include maternally inherited Leigh syndrome and neurogenic muscle weakness, ataxia, and retinitis pigmentosa (NARP). The presentation and severity of these are usually dependent on the level of mutant mtDNA (heteroplasmic load) in different tissue types.^3^ Recently, the clinical spectrum of mitochondrial ATP synthase disorders has expanded further to include axonal Charcot-Marie-Tooth disease,^4^ late-onset hereditary spastic paraplegia-like disorder,^5^ and episodic weakness.^6^ The majority of *MT-ATP6* mutations are missense; only 3 truncating mutations are reported, all of which presented with ataxia, developmental delay, or NARP.^7–9^

Here, we describe 3 patients harboring heteroplasmic truncating *MT-ATP6* mutations; 2 harboring a novel de novo variant and a third with a maternally inherited, previously reported, mutation. The structural and functional consequences of both mutations in all the 3 patients are presented.

## Methods

### Standard protocol approvals, registrations, and patient consents

The study was performed under the ethical guidelines issued by the relevant local ethical committees of the participating centers with written informed consent obtained from participants.

### Patient 1

**Figure 1:**
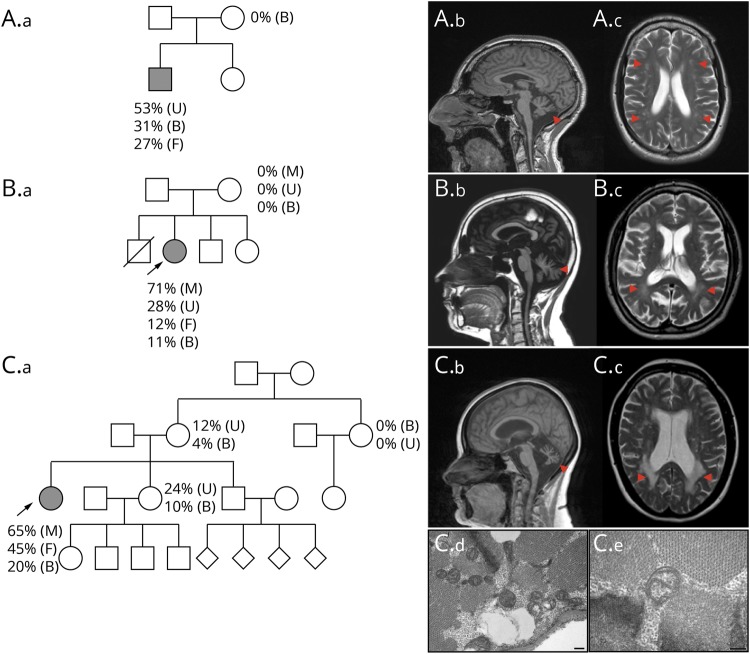
Pedigrees and brain MRI findings (A.a) Family pedigree chart of patient 1 (P1) harboring m.8782 G>A; p.(Gly86*) mutation. Brain MRI showing cerebellar atrophy (red arrowhead, A.b) and multiple deep and periventricular white matter changes (red arrowheads, A.c). (B.a) Family pedigree chart of patient 2 (P2) harboring m.8782G>A; p.(Gly86*) mutation. Brian MRI showing reduced brain volume, marked global cerebellar, and brainstem atrophy (red arrowhead, B.b) and multiple deep and periventricular white matter changes (red arrowheads, B.c). (C.a) Family pedigree chart of patient 3 (P3) harboring m.8618dup; p.(Thr33Hisfs*32) mutation. Brain MRI showing severe cerebellar atrophy (red arrowhead, C.b) and posterior white matter abnormalities (red arrowheads, C.c). Electron microscopy showing several mitochondria with simplified internal structure (C.d). High magnification (C.e) showing a mitochondria with aberrant cristae formation. Scale bar represents 200 nm. Filled symbols indicate affected individuals. Symbols with diagonal strikethrough indicate deceased. Arrows indicate probands. Mutation load detectable in different tissues: B = blood; F = fibroblasts; M = muscle; U = urine.

The proband (P1), a 37-year-old man, is the eldest of 2 siblings from nonconsanguineous parents. Intrauterine growth restriction was reported, but early motor development was otherwise normal. At 10 years of age, growth hormone replacement was commenced for short stature. He subsequently developed noninsulin-dependent diabetes at the age of 24 years and was diagnosed with focal segmental glomerulosclerosis 1 year later. He subsequently developed imbalance (28 years), sensorineural hearing loss (30 years), impaired exercise tolerance and muscle aches/cramps (34 years), and complex partial seizures (36 years). There is no family history; both parents and his 27-year-old sister are healthy (figure 1A). Clinical examination at the age of 36 years revealed short stature (5 feet 5 inches), microcephaly, a mild head tremor, an ataxic gait, bilateral sensorineural hearing loss, and impaired coordination. There were upper motor neuron signs in the limbs, with increased tone and pathologically brisk reflexes. Blood lactate at the age of 35 years was elevated (4.66 IU/L, reference range 0.5–2.2). Nerve conduction studies and EMG showed no evidence of neuropathy or myopathy. EEG was normal. Brain MRI showed left sided mesial temporal sclerosis, cerebellar atrophy, and white matter changes (figure 1A). Diagnostic next generation sequencing (NGS) of mtDNA in blood confirmed the novel heteroplasmic truncating *MT-ATP6* variant m.8782G>A; p.(Gly86*). Mutant m.8782G>A; p.(Gly86*) levels varied across the tissues, with 31% mutant load detected in blood leucocytes, 53% in urinary epithelial cells, and 27% in primary fibroblasts. The variant was undetectable in mtDNA extracted from the blood leucocytes of the P1's asymptomatic mother (figure 1A).

### Patient 2

The proband (P2) is a 38-year-old woman born after uncomplicated pregnancy and delivery from Italian nonconsanguineous parents. Cognitive impairment was reported in late infancy. At 25 years of age, she had frequent episodic jerks and tremors of the arms and legs and was diagnosed with myoclonic epilepsy at the age of 32 years. At 36 years of age, she underwent bilateral cataract surgery. She had also developed gait instability, speech impairment, hearing loss, and tinnitus. Her mother was reported to have migraine, focal-onset epilepsy with a normal brain MRI, type 2 diabetes, and lipomas. The proband was the second born of 4 children; the eldest brother died at 3 months of age for a not specified cardiomyopathy. Both her 31-year-old brother and 16-year-old sister are healthy (figure 1B). Clinical examination of the proband at the age of 38 years showed short stature, hypertrichosis, dysarthria, nystagmus in lateral gaze, upper limb tremor, dysmetria, and ataxic gait. ECG and echocardiogram were normal. Pure tone audiometry confirmed sensorineural hearing loss. EEG confirmed epileptic myoclonus, and no myopathic changes were found in the EMG. Brain CT showed basal ganglia calcification. Brain MRI performed at the age of 38 years demonstrated cerebellar atrophy, brainstem atrophy, and mild white matter abnormalities (figure 1B), and spectroscopy detected a peak of lactate in the cerebellum voxel. Muscle biopsy showed mild myopathic features with a single cytochrome *c* oxidase (COX) deficient ragged red fiber (RRF). Spectrophotometric assays of mitochondrial respiratory chain (RC) complexes I–IV in muscle and complexes I–V in fibroblast, normalized for citrate synthase (CS) activity, was normal.

NGS of the entire mitochondrial genome detected the m.8782G>A: p.(Gly86*) variant in *MT-ATP6*; it was present at 71% mutant load in skeletal muscle tissue, 11% in blood, 28% in urinary epithelial cells, and 12% in fibroblasts. The mutation was considered de novo in the proband, given that it was undetectable in maternal blood lymphocytes, urinary epithelial cells, and skeletal muscle tissue.

### Patient 3

The proband (P3), a 42-year-old woman, was the eldest of 3 siblings from nonconsanguineous parents. She was born at term without any pregnancy complications. She presented to pediatric medical services in early infancy with failure to thrive, because of feeding difficulties, and global developmental delay; she walked at 18 months, but gait was poorly coordinated, and she was never able to keep up with peers when running. During adolescence, she developed scoliosis, bilateral cataracts, tapetoretinal degeneration, and mild aortic regurgitation. At 23 years of age, she was diagnosed with learning difficulties and Asperger syndrome. Sensorineural deafness was detected at the age of 30 years, followed by chronic kidney disease, which was treated initially with hemodialysis, followed by a renal transplant, at the age of 31 years. At 32 years of age, she had surgery for bilateral cataracts and developed diabetes at 38 years, which is now managed with insulin. More recently, she has developed dysphagia and cognitive decline. Owing to progressive imbalance, she now requires a wheelchair for long distances. There was no family history for neurologic or multisystem disease (figure 1C). Clinical examination at the age of 36 years confirmed an ataxic, spastic gait and short stature (4 feet 8 inches). Cranial nerve examination revealed reduced upgaze, dysarthria, and slow tongue movements. In the limbs, there was a mild bilateral postural tremor, increased tone, and brisk reflexes with normal sensation. Blood lactate was mildly elevated (2.59 mmol/L, reference range 0.5–2.2). Brain MRI showed white matter changes and cerebellar atrophy (figure 1C). Muscle biopsy showed mild myopathic features and increased lipid content with no RRF or COX deficient fibers. Electron microscopy (EM) showed several, scattered mitochondria with abnormalities in the cristae (figure 1C). Spectrophotometric assays of mitochondrial RC complexes I and IV in the muscle, corrected for CS, was normal. Enzyme assay of complex II + III (succinate: cytochrome *c* reductase) revealed reduced activity (complex II + III/CS ratio 0.018, reference range 0.04–0.204). However, subsequent analysis of complex II and muscle ubiquinone was normal (complex II/CS 0.097, reference range 0.052–0.25, ubiquinone 237 pmol/mg, reference range 140–580 pmol/mg). Array comparative genomic hybridization analysis was normal. NGS of mtDNA confirmed the previously reported heteroplasmic truncating *MT-ATP6* mutation, m.8618dup; p.(Thr33Hisfs*32).^7^ Mutant m.8618dup; p.(Thr33Hisfs*32) levels were confirmed across multiple tissues. These included 20% in blood leucocytes, 45% in primary fibroblasts, and 65% in muscle tissue. The mutation was detected in other unaffected family members at substantially lower levels (figure 1C).

After identification of the 2 heteroplasmic truncating *MT-ATP6* mutations, experiments to confirm their downstream pathologic effects on mitochondrial ATP synthase structure and function were undertaken, using patient-derived mutant fibroblasts (P1 and P3), transmitochondrial cybrids (obtained from mutant fibroblast P2) and muscle samples (P2 and P3).

### Cell culture

Primary fibroblast cultures were obtained from healthy controls and patients (P1, P2, and P3). Human fibroblasts were grown either in Dulbecco's Modified Eagle Medium with 4.5 g/L glucose, 10% fetal calf serum, 1 mM sodium pyruvate, 200 U/mL penicillin G, and 200 mg/mL streptomycin or in an equivalent medium in which glucose was replaced by 5 mM galactose, at 37°C in a humidified 5% CO_2_ atmosphere. Cybrids were obtained from P2 as previously described^10,11^ (e-methods 1 and figure e-1, links.lww.com/NXG/A201).

### Blue-native gel electrophoresis

Blue-native gel electrophoresis (BNGE) on fibroblast (P1 and P3) and muscle (P2 and P3) samples was performed as previously described.^12,13^ Mitochondria were solubilized with either n-dodecyl-β-d-maltoside (DDM), 1.6 mg/mg of mitochondrial protein or digitonin 4 mg/mg of mitochondrial protein.^13^ Samples were run on precast native polyacrylamide 3%–12% Bis-Tris gels. Proteins were either transferred on a nitrocellulose membrane (1D-BNGE) or denatured and run on SDS-PAGE (2D-BNGE). Anti-*ATP5A* (Abcam, ab14748), anti-*ATP6* (Abcam ab219825), and anti-COXIV (Abcam, ab14744) antibodies were used for complex V and complex IV visualization.

### Western blot

Mitochondrial-enriched fractions from the muscle (P2 and P3) were separated by denaturing NuPAGE 4%–12% Bis-Tris gels and blotted with anti-*ATP6* (Abcam ab219825) and anti-VDAC1/porin (Abcam, ab154856) antibodies.

### Microscale oxygraphy

The oxygen consumption rate (OCR) was measured in adherent fibroblasts (P1 and P3) with a XF96 Extracellular Flux Analyzer (Seahorse Bioscience, Billerica, MA). Control and mutant fibroblast cell lines were seeded in 8–12 wells of a XF 96-well cell culture microplate (Seahorse Bioscience) at a density of 15–20 × 10^3^ cells/well and analyzed after 24 hours. OCR was measured at baseline and after sequentially adding of 1 μM oligomycin A, 1 μM of carbonyl cyanide 4-(trifluoromethoxy) phenylhydrazone, and 1 μM of rotenone and antimycin A (protocol available on request). Normalization was performed with a CyQUANT Cell Proliferation Assay Kit (Thermo Fisher Scientific, cat. No. C35007), according to the manufacturer's instructions.

### Reactive oxygen species measurement

Reactive oxygen species (ROS) were measured on patients' fibroblasts (P1 and P3). Cells were seeded in 15–20 × 10^3^ cells/well in 96-well plate and used after 24 hours. Cells were washed with PBS and incubated for 30 minutes with 100 μM of CM-H2DCFDA (Thermo Fisher Scientific, cat. N. C6827), and then, fluorescence was recorded for 30 minutes with Ex 488/Em 525 nm. Equal numbers of cells were seeded in a separate 96-well plate and normalization was performed as previously mentioned.

### Statistical analysis

All numerical data are expressed as mean ± SEM. After assessment for normality Student unpaired 2-tail *t* test was used for statistical analysis of the OCR data, whereas paired *t* test was used for statistical analysis of the ROS data. Differences were considered statistically significant for *p* ≤ 0.05.

### Data availability

The data that support the findings of this study are available on request from any qualified investigator.

## Results

### Deleterious effects of the m.8782G>A mutation in cybrids with high mutant load

The RC activity on fibroblasts in 12% mutated clones was normal for all complexes (CI-CV). The RC on transmitochondrial cybrids in 0% and in 10% mutated clones was normal while a severe reduction of complex V was revealed in the 95% mutated clone (29.10, 28.90, and 8.30 nmol/min per mg of protein for 0%, 10%, and 95% mutated cybrids, respectively).

### Impaired complex V assembly

**Figure 2:**
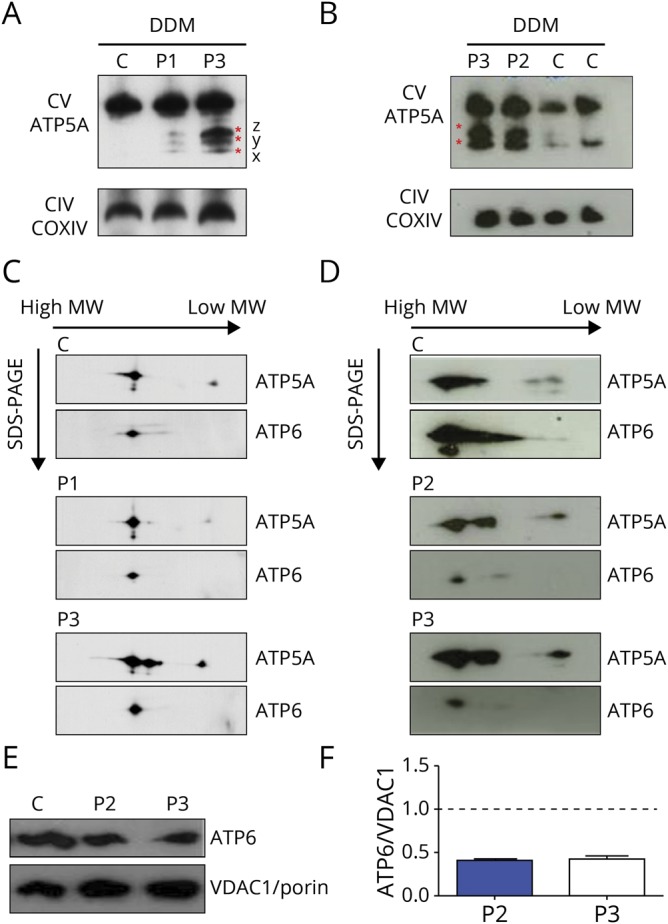
One-dimensional and 2-dimensional BNGE (A and B) Immunovisualization of complex V in 1-dimensional BNGE of enriched mitochondria fractions extracted from fibroblasts and muscles and solubilized with DDM. Control (C), patient 1 (P1), patient 2 (P2), and patient 3 (P3) are shown. Three F1 x, y, and z subcomplexes are present in fibroblasts of P1 and P3, whereas only 2 of 3 subassemblies are present in the muscle of P3 and P2. See discussion for details. Anti-*ATP5A* and anti-COXIV used to visualize complex V and complex IV, respectively. (C and D) Denaturing 2-dimensional BNGE of enriched mitochondria fractions extracted from fibroblasts and muscle and solubilized with DDM confirmed the presence of ATP synthase subcomplexes in P1, P2, and P3. Residual *ATP6* protein is found incorporated into the fully assembled complex V in P1, P2, and P3. (E) Western blot of muscle samples show reduced *ATP6* protein in patients (P2, P3) compared with the control. (F) Densitometry analysis of (E) performed in 2 different experiments. Values are normalized to controls. Error bars represent SEM. ATP = adenosine triphosphate; BNGE = blue-native gel electrophoresis; CIV = complex IV; COXIV = cytochrome *c* oxidase IV; CTR = control; CV = complex V; DDM = n-dodecyl-β-d-maltoside; MW = molecular weight; SDS-PAGE = sodium dodecyl sulfate-polyacrylamide gel electrophoresis; SEM = standard error of the mean; VDAC1 = voltage dependent anion channel 1.

Complex V assembly defect was present in DDM-solubilized mitochondria from both the patient-derived fibroblasts (P1 and P3); according to the mutant load, P3 was more severely affected than P1 (45% vs 27%, figure 2A). Mutations in the *ATP6* gene resulted in accumulation of F1 subcomplexes (x, y, and z)^14^ that were detectable using an antibody to *ATP5A*, the early assembled ATP synthase F1 subunit α (figure 2A). Complex intermediates were also demonstrated in muscle samples (P2 and P3, figure 2B). Notably, fully assembled complex V was detectable in patients and 2D-BNGE confirmed that residual *ATP6* protein was indeed incorporated into complex V (figure 2, C and D). Similar results were obtained in samples solubilized with digitonin (not shown). *ATP6* levels in the 2D BNGE performed in the muscle were reduced compared with the control (figure 2D) in agreement with the mutant load found in this tissue (71% in P2 and 65% in P3). We evaluated the steady state level of *ATP6* protein in muscle samples, and we confirmed a reduction of ≈40% in both patients (figure 2, E and F).

### Reduced basal respiration and ATP synthesis

**Figure 3:**
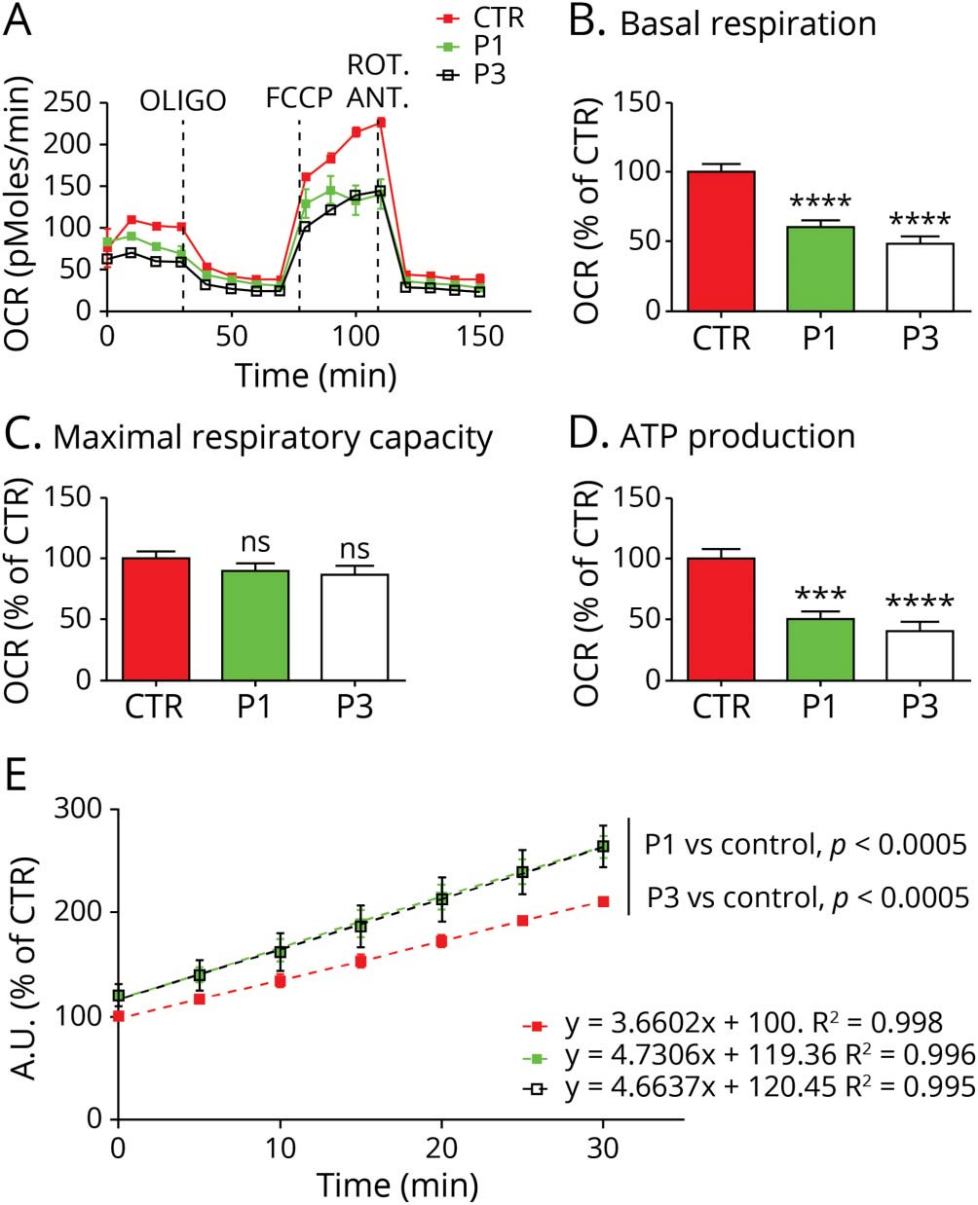
Microscale oxygraphy and reactive oxygen species measurement (A) Representative graph illustrating the protocol used to measure mitochondrial respiration in fibroblasts using a XF96 extracellular flux analyzer (Seahorse Bioscience). The data represent the outcome of an experimental run before normalization. Bar charts showing (B) basal respiration, (C) maximal Respiratory capacity, and (D) ATP production. Data are the average of 3 biological replicates, n = 30–50 measurements. OCR were normalized to the number of cells and expressed as percentage of control. Error bars represent SEM, ***p < 0.0005, ****p < 0.0001 by unpaired Student *t* test. (E) Linear regression of the time course analysis of reactive oxygen species production measured by DCFDA in cultured fibroblasts. Error bars represent SEM, statistical analysis performed by paired *t* test. Color code as in A. ANT = antimycin; FCCP = carbonyl cyanide 4-(trifluoromethoxy) phenylhydrazone; ns = nonsignificant; OCR = oxygen consumption rates; OLIGO = oligomycin; ROT = rotenone; SEM = standard error of the mean.

Both P1 and P3 mutant fibroblasts demonstrated reduced OCR and ATP production in basal conditions (Figure 3, A–D). The latter was more pronounced in the fibroblasts of P3, which harbored higher *MT-ATP6* mutation levels (27% and 45% mutant load in P1 and P3, respectively). Maximal respiratory capacity was not statistically different in control when compared with mutant fibroblasts (figure 3C).

### Increased reactive oxygen species

ROS production was significantly higher (+19% P1, *p* < 0.01 and +20% P3, *p* < 0.05, at T = 0, figure 3E), and the rate of ROS production (measured over 30 minutes) was greater in the patient-derived fibroblasts compared with the controls (4.66 A.U./min P1 and 4.73 A.U./min P3 vs 3.66 A.U./min control, *p* < 0.0005 for P1 and P3, figure 3E). There was no significant difference between the level of ROS produced between P1 and P3 (*p* = 0.1039).

### Mitochondrial morphology

Mitochondrial morphology in the cultured fibroblasts of P1 and P3 was normal (e-methods and figure e-2, links.lww.com/NXG/A201).

## Discussion

These data confirm the pathogenic effects of 1 novel (m.8782G>A) and 1 previously reported (m.8618dup) heteroplasmic truncating mutation in *MT-ATP6*. The frameshift caused by the m.8618dup results in a premature downstream stop codon (TAA) ^7^ while the m.8782G>A mutation changes GGA to AGA, the structural consequences of which are thought to cause either a stop codon or a ribosomal frameshift-1.^15^ Assuming the ribosomal frameshift mechanism, a stop codon 31 amino acids downstream to the mutation is predicted, thereby creating a truncated protein. We also expand the clinical phenotypic spectrum of mitochondrial ATP synthase disorders to include (1) leukodystrophy and cognitive decline, with predominant posterior white matter abnormalities, and severely impaired kidney function requiring renal transplantation caused by m.8618dup; p.(Thr33Hisfs*32) and (2) myoclonic epilepsy with ataxia associated with m.8782G>A; p.(Gly86*).

The majority of mitochondrial *ATP6* synthase disorders result from missense mutation in *MT-ATP6*; moderate mutant levels (70%–90%) present with early onset ataxia, learning difficulties, and retinal involvement while patients with higher mutant loads (>90%) develop maternally inherited Leigh syndrome. Significant clinical overlap exists between the 4 previously and 3 newly reported patients harboring truncating *MT-ATP6* mutations and those with missense *MT-ATP6* variants. However, additional features, including cerebral white matter changes and renal impairment (4 of 6 and 3 of 6 patients with data available exhibited these clinical manifestations, table), are reported more frequently.^7–9,16^ White matter abnormalities are not uncommon in Leigh syndrome^17^; however, ATP synthase dysfunction has been reported with this clinic-radiologic association infrequently^17,18^ while chronic kidney disease with isolated proteinuria, which did not require renal replacement therapy, has been reported in a single case of NARP.^19^ P2 presented with myoclonic epilepsy with RRF (MERRF)-like phenotype, typically associated with the m.8344A>G in *MT-TK*. Of interest the previously reported truncating *MT-ATP6* mutation m.9127-9128delAT; p.(Ile201Profs*2) was associated with myoclonic epilepsy, ataxia, and cerebellar atrophy, although the muscle biopsy did not reveal RRF.^9^ The phenotype was considered consistent with NARP, despite no evidence of neuropathy. Consequently, we suggest truncating *MT-ATP6* mutations should be excluded in patients presenting with a MERRF-like phenotype if genetic analysis for more well-recognized causes, such as the m.8344A>G mutation, are negative. Muscle histopathology is generally unhelpful when diagnosing mitochondrial ATP synthase disorders caused by missense mutations in *MT-ATP6/8*, and this also applies to *MT-ATP6* truncating mutations (table). Of note, EM demonstrated simplified cristae structure in the muscle of P3, a finding reported in the fibroblasts of a patient harboring the truncating *MT-ATP6* mutation m.8611insC; p.(Pro29Leufs*36).^8^ This observation potentially relates to the physiologic role that ATP synthase plays in cristae formation.^20^

**Table:**
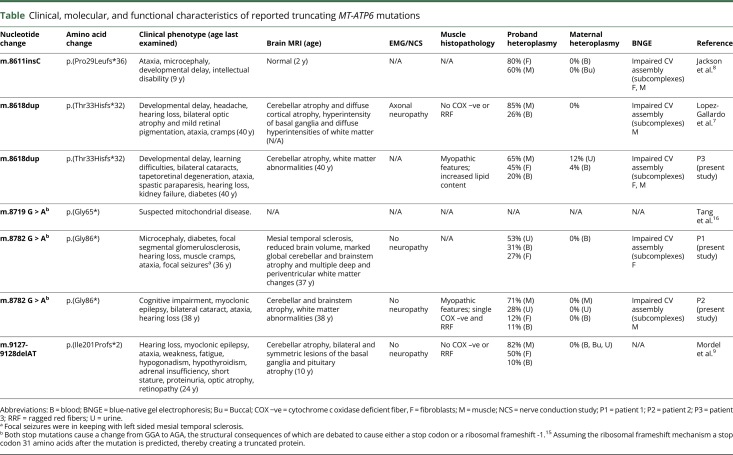
Clinical, molecular, and functional characteristics of reported truncating *MT-ATP6* mutations

One further important consideration when counseling patients with truncating *MT-ATP6* mutations is that mutant load varies considerably across different tissues unlike the most common *MT-ATP6* missense mutations.^3,4^ As such, the level of mutation detectable in blood and/or muscle may not be representative of peripheral and central nervous tissue, a factor that potentially explains the discordant clinical syndromes associated with the m.8618dup; p.(Thr33Hisfs*32) mutation: spastic tetraparesis, posterior leukodystrophy, cognitive decline, and severe renal disease in P3, and a previously reported case of NARP.^7^ Furthermore, given the variability in mutant load tissue observed for the *MT-ATP6* truncating mutations, accurate correlation between heteroplasmy levels and disease severity was not possible. Finally, blood m.8618dup mutant levels were low in both cases: 11% blood vs 71% muscle (P3) and 26% blood vs 85% muscle.^7^ These data highlight the importance of undertaking molecular analysis in multiple tissue types, given the variable segregation truncating *MT-ATP6* mutations exhibit.

Patient-derived fibroblasts (P1 and P3) and muscle sample (P2 and P3) were used to demonstrate the downstream effects of the m.8782G>A; p.(Gly86*) and m.8618dup; p.(Thr33Hisfs*32) mutations on complex V assembly and OXPHOS. BNGE analysis of mitochondria from patients' fibroblasts confirmed the presence of the F1 subcomplexes (x, y, and z) that were previously reported in Rho^0^ cells, patient-derived mutant *MT-ATP6* cell lines, and mtDNA depletion syndrome (figure 2A).^4,14^ The presence of fully assembled complex V, with detectable *ATP6*, in the patients' fibroblasts and skeletal muscle, is consistent with the heteroplasmic state of the *MT-ATP6* mutations (P1 27%, P3 45% mutant loads in fibroblasts; P2 71% and P3 65% in the muscle; figure 2, C and D). The level of *ATP6* protein incorporated into complex V was reduced compared with the control in muscle samples (figure 2D). When steady state of *ATP6* protein in muscle sample was evaluated, we confirm the reduction of *ATP6* protein (figure 2E) as reported in a previous case with the same mutation found in P3.^7^ Cybrid cell lines confirmed the deleterious effect of the new m.8782G>A mutation, while microscale oxygraphy demonstrated that both mutations reduce basal respiration and ATP synthesis and increase ROS production, findings previously reported with *MT-ATP6* mutations.^21,22^ This bioenergetic profile is consistent with impaired ATP synthase activity and is recapitulated using oligomycin treatment in wild-type cells.^23^ Reduced complex V activity is also linked to an increase in mitochondrial membrane potential. This stimulates an electron leak within the RC and generates high ROS levels.^23,24^ It is possible that this mechanism is also contributing to the high ROS levels exhibited by the fibroblasts of P1 and P3. Finally, maximal respiration capacity, as determined by the mitochondrial electron transport chain enzymes (complexes I–IV), was retained in the mutant cell lines, consistent with an isolated defect of ATP synthase function. Reduced maximum respiratory capacity is reported in the mutant cell lines harboring high levels of m.8993T>C/G.^25^ However, this is partly explained by the presence of additional mtDNA variations that compounded the RC defect, as shown in cybrids models.^26^ The relatively low *MT-ATP6* mutant levels, or possibly an efficient background bioenergetic profile, might account for the normality of the respiratory capacity in our patient-derived fibroblasts.

We expand the molecular and phenotypic spectrum of mitochondrial *ATP6* synthase disorders by reporting the clinicoradiological, structural, and functional characteristics of 1 novel and 1 maternally inherited heteroplasmic truncating *MT-ATP6* mutation and highlight the variable tissue segregation of these variants, which should be considered when counseling patients.

## Glossary

BNGE: blue-native gel electrophoresis
CMT: Charcot-Marie-Tooth
COX: cytochrome c oxidase
CS: citrate synthase
EM: electron microscopy
MERRF: myoclonic epilepsy with RRF
MILS: maternally-inherited Leigh syndrome
mtDNA: mutations in mitochondrial DNA
NARP: neurogenic muscle weakness, ataxia and retinitis pigmentosa
NCS: nerve conduction study
NGS: next generation sequencing
OCR: oxygen consumption rate
OXPHOS: oxidative phosphorylation
RC: respiratory chain
ROS: reactive oxygen species
RRF: ragged red fiber
